# ADAPT NXT: Fixed Cycles or Every‐Other‐Week IV Efgartigimod in Generalized Myasthenia Gravis

**DOI:** 10.1002/acn3.70051

**Published:** 2025-04-14

**Authors:** Ali A. Habib, Kristl G. Claeys, Vera Bril, Yessar Hussain, Kelly Gwathmey, Gregory Sahagian, Elena Cortés‐Vicente, Edward Brauer, Deborah Gelinas, Anne Sumbul, Rosa H. Jimenez, Daniela Hristova, Delphine Masschaele, Renato Mantegazza, Andreas Meisel, Shahram Attarian, the ADAPT NXT Study Group

**Affiliations:** ^1^ Department of Neurology University of California, Irvine Orange California USA; ^2^ Department of Neurology University Hospitals Leuven Leuven Belgium; ^3^ Laboratory for Muscle Diseases and Neuropathies KU Leuven Leuven Belgium; ^4^ Ellen & Martin Prosserman Centre for Neuromuscular Diseases University Health Network Toronto Ontario Canada; ^5^ University of Toronto Toronto Ontario Canada; ^6^ Austin Neuromuscular Center Austin Texas USA; ^7^ Department of Neurology Virginia Commonwealth University Richmond Virginia USA; ^8^ The Neurology Center of Southern California Carlsbad California USA; ^9^ Neuromuscular Diseases Unit, Department of Neurology Hospital de la Santa Creu i Sant Pau Barcelona Spain; ^10^ Biomedical Research Institute Sant Pau Barcelona Spain; ^11^ Centro de Investigación Biomédica en Red de Enfermedades Raras, CIBERER Valencia Spain; ^12^ argenx Ghent Belgium; ^13^ Emeritus Fondazione IRCCS Istituto Neurologico Carlo Besta Milan Italy; ^14^ Department of Neurology With Experimental Neurology and NeuroScience Clinical Research Center Charité – Universitätsmedizin Berlin Berlin Germany; ^15^ Reference Center for Neuromuscular Disorders and ALS Timone Hospital University Marseille France

**Keywords:** efgartigimod, generalized myasthenia gravis, myasthenia gravis activities of daily living, neonatal Fc receptor

## Abstract

**Objective:**

This phase 3b, open‐label, randomized ADAPT NXT study investigated the efficacy, safety, and tolerability of efgartigimod administered in either a fixed cycles dosing regimen (3 cycles of 4 once‐weekly infusions, with 4 weeks between cycles) or a cycle followed by every‐other‐week (Q2W) dosing.

**Methods:**

Adult participants with anti‐acetylcholine receptor antibody‐positive generalized myasthenia gravis (gMG) were randomized 3:1 to Q2W or fixed cycles dosing of efgartigimod (10 mg/kg intravenously) for 21 weeks. The primary endpoint was the mean change from baseline in total Myasthenia Gravis Activities of Daily Living (MG‐ADL) score averaged across 21 weeks.

**Results:**

Sixty‐nine participants were treated (fixed cycles, *n* = 17; Q2W, *n* = 52). Least squares (LS) mean (95% CI) of the change from baseline in MG‐ADL total score from Weeks 1 to 21 was −5.1 (−6.5 to −3.8) in the fixed cycles arm and −4.6 (−5.4 to −3.8) in the Q2W arm. Clinical improvements were observed in MG‐ADL total scores as early as Week 1 and were maintained throughout the study. Achievement of minimal symptom expression (MG‐ADL: 0–1) from Weeks 1 to 21 occurred in 47.1% (*n* = 8/17) and 44.2% (*n* = 23/52) of participants in the fixed cycles and Q2W arms, respectively. Efgartigimod was well tolerated; COVID‐19, headache, and upper respiratory tract infection were the most common treatment‐emergent adverse events.

**Interpretation:**

Efgartigimod administered as either fixed cycles or Q2W dosing results in rapid, robust, and sustained clinically meaningful improvement. These results build upon previous studies and provide additional efgartigimod dosing approaches to achieve and sustain clinical efficacy in patients with gMG.

## 1 Introduction

Generalized myasthenia gravis (gMG) is an immunoglobulin G (IgG)–mediated autoimmune disease characterized by autoantibodies targeting components of the neuromuscular junction [1]. Pathogenic IgG autoantibodies, detectable in approximately 85%–90% of patients with gMG, bind postsynaptic proteins of the neuromuscular junction including acetylcholine receptor (AChR), muscle‐specific tyrosine kinase (MuSK), and low‐density lipoprotein receptor‐related protein 4 (LRP4) [1, 2, 3]. These autoantibodies impair neuromuscular transmission through multiple mechanisms, including functional blockade and cross‐linking/internalization of AChR, as well as complement activation, resulting in fatigable weakness of voluntary muscles [4, 5, 6]. Myasthenia gravis often manifests initially in ocular muscles (ocular MG; oMG) and can progress to gMG with involvement of bulbar, axial, limb, and/or respiratory muscles resulting in 15%–20% of patients experiencing potentially life‐threatening myasthenic crisis in their lifetime [7, 8]. Broadly acting immunosuppressives (including corticosteroids and nonsteroidal immunosuppressive therapies [NSISTs]) reduce morbidity and mortality, but patients often experience residual symptoms [6, 8, 9, 10, 11]. Moreover, the generalized immunosuppressive actions of these therapies may contribute to adverse events and subsequent comorbidities [6, 10, 11, 12, 13]. Complement inhibitors address only one of the three pathogenic pathways triggered by autoantibodies in gMG, and require vaccination and/or antibiotic prophylaxis [5, 14, 15]. Accordingly, an effective treatment approach that is well tolerated and targets all autoantibody‐driven pathologies of gMG may address unmet therapeutic needs [6, 16, 17].

The neonatal Fc receptor (FcRn) recycles IgG antibodies and albumin by saving them from lysosomal degradation [18]. This recycling by FcRn results in IgG antibodies having the longest half‐life and being the most abundant of all immunoglobulins [5, 19]. In patients with IgG‐mediated autoimmune diseases, including gMG, recycling by FcRn prolongs the availability of IgG autoantibodies that are central to disease pathology [2, 18]. Blocking FcRn to selectively reduce IgG levels through lysosomal degradation of unbound IgG is therefore a rational therapeutic approach in patients with IgG‐mediated autoimmune diseases [2, 16, 18].

Efgartigimod is an IgG1 antibody Fc fragment, uniquely composed of only the part of the IgG antibody that naturally binds FcRn, which has been engineered for increased affinity to FcRn [2, 16]. As an Fc antibody fragment that binds FcRn in the same way as endogenous IgG, efgartigimod's molecular design ensures it does not reduce albumin levels, increase cholesterol levels, or affect cellular trafficking of FcRn [2, 20, 21]. Efgartigimod selectively reduces IgG levels (including all IgG subtypes) by blocking FcRn‐mediated IgG recycling, without impacting antibody production or other parts of the immune system [2, 22, 23]. It prevents IgG recycling by blocking IgG antibodies from binding to FcRn, with unbound IgG antibodies then being degraded [2, 16]. Across multiple dosing regimens, exposure times, and formulations (intravenous [IV] and subcutaneous [SC]) employed in both healthy volunteers and participants with various IgG‐mediated autoimmune disorders, efgartigimod was well tolerated and led to a ~60%–70% decrease in total IgG levels [2, 16, 24, 25, 26, 27, 28, 29]. Efgartigimod has been studied in more than 2000 healthy volunteers and patients with various IgG‐mediated autoimmune disorders (manuscript in preparation), and is currently approved in multiple countries for the treatment of gMG [30]. It is also indicated in the United States for the treatment of chronic inflammatory demyelinating polyneuropathy (CIDP), in Japan for the treatment of primary immune thrombocytopenia (ITP), and is being studied in many other IgG autoantibody‐mediated autoimmune diseases [31, 32, 33].

Efgartigimod was shown to be effective and well tolerated in participants with acetylcholine receptor antibody‐positive (AChR‐Ab+) gMG in the ADAPT and ADAPT‐SC clinical trials, and the long‐term safety and efficacy profile were established during the associated open‐label extensions (ADAPT+ [completed] and ADAPT‐SC+ [completed]) [16, 27, 34]. In these studies, efgartigimod was administered in cycles of either 4 once‐weekly IV infusions or 4 once‐weekly SC injections of efgartigimod PH20 (coformulated with recombinant human hyaluronidase PH20). As anticipated due to the heterogeneity of the disease, the frequency and number of cycles varied between individuals, with some participants exhibiting extended clinical benefit while other participants required more frequent cycles of efgartigimod [27, 34].

In order to extend the options for individualization of efgartigimod dosing, the objective of the phase 3b, randomized, open‐label ADAPT NXT study was to assess the efficacy, safety, and tolerability of IV efgartigimod administered in two additional dosing regimens. These regimens included initiating treatment using a fixed cycles dosing regimen (3 cycles of 4 once‐weekly infusions, with 4 weeks between cycles) or a cycle of 4 once‐weekly infusions followed by every‐other‐week (Q2W) dosing, which were not explicitly included in previous clinical trials of participants with gMG.

## 2 Materials and Methods

### 2.1 Study Design

**FIGURE 1:**
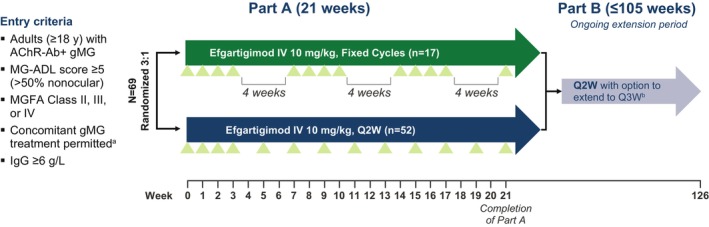
Study design. Green triangles indicate efgartigimod infusion. ^a^Including NSISTs, corticosteroids, and/or AChEIs. If receiving corticosteroids and/or NSISTs, must be on a stable dose for ≥ 1 month prior to screening. ^b^All participants entering Part B will be transitioned to Q2W with the option to extend to Q3W dosing based on clinical judgment and guided by the MG‐ADL total score. AChEI, acetylcholinesterase inhibitor; AChR‐Ab+, acetylcholine receptor autoantibody positive; gMG, generalized myasthenia gravis; IgG, immunoglobulin G; IV, intravenous; MG‐ADL, Myasthenia Gravis Activities of Daily Living; MGFA, Myasthenia Gravis Foundation of America; NSIST, nonsteroidal immunosuppressive therapy; Q2W, every other week; Q3W, every third week.

ADAPT NXT (NCT04980495) is a phase 3b, randomized, open‐label, parallel‐arm study evaluating two dosing regimens of efgartigimod IV (10 mg/kg). Participants were recruited across 31 sites in Europe and North America. During the 21‐week Part A phase of the study, participants were randomized 3:1 to either a Q2W dosing regimen or a fixed cycles dosing regimen (Figure 1). This 3:1 randomization ratio was selected because cyclic dosing has been well studied in > 150 participants with gMG during ADAPT and ADAPT+ and > 175 participants during ADAPT‐SC and ADAPT‐SC+ [16, 27, 34]. In these previous studies, the timing of subsequent treatment cycles was not fixed, and treatment was administered according to clinical evaluation. In the fixed cycles dosing regimen of ADAPT NXT, participants received 3 cycles of 4 once‐weekly IV infusions of efgartigimod, with 4 weeks between cycles. In the Q2W dosing regimen, efgartigimod was administered as 1 cycle of 4 once‐weekly infusions, followed by every‐other‐week dosing through Week 21 of the study. In the ongoing Part B of ADAPT NXT, all participants receive efgartigimod IV infusions Q2W, with the option to extend dosing frequency to every 3 weeks, depending on clinical evaluation and guided by the Myasthenia Gravis Activities of Daily Living (MG‐ADL) score of each participant. ADAPT NXT was conducted in accordance with the Declaration of Helsinki and Council for International Organizations of Medical Sciences international ethical guidelines, applicable International Council for Harmonisation of Technical Requirements for Pharmaceuticals for Human Use Good Clinical Practice guidelines, and other applicable local laws and regulations. The protocol and informed consent forms were submitted to and approved by independent review boards and/or independent ethics committees.

### 2.2 Participants

Participants were eligible to enroll in the study if they were ≥ 18 years of age with AChR‐Ab+ gMG and had an MG‐ADL total score of ≥ 5, with > 50% of the score due to nonocular symptoms. They were required to meet the clinical criteria for Myasthenia Gravis Foundation of America (MGFA) class II, III, or IV. If patients were receiving concomitant gMG treatment, a stable dose of therapy was required through the end of Part A; NSISTs and corticosteroids (on stable dose for ≥ 1 month before screening), as well as acetylcholinesterase inhibitors (AChEIs), were permitted.

Participants were excluded if they had previously been treated with efgartigimod, their total IgG was < 6 g/L, or if they had a thymectomy within 3 months of screening. Additionally, participants were excluded if they had received IV or subcutaneous immunoglobulin (SCIg) within 2 weeks, eculizumab within 1 month, or rituximab within 6 months of study Day 1, or live/live‐attenuated vaccines within 1 month prior to screening. Full inclusion and exclusion criteria are reported in Table S1.

### 2.3 Procedures

The primary endpoint was the mean change from baseline in total MG‐ADL score averaged across 21 weeks. Additional efficacy endpoints included change from baseline in the MG‐ADL total score throughout the study (assessed weekly) and percentage of participants achieving minimal symptom expression (MSE; defined as an MG‐ADL total score of 0–1) through Weeks 1 to 21 of the study. Safety was assessed through incidence and severity of adverse events (continuously monitored), as well as changes in laboratory values, vital signs, and electrocardiograms (monitored on Day 1 and at Weeks 4, 7, 14, and 21). The severity of adverse events was graded using the National Cancer Institute Common Terminology Criteria for Adverse Events (version 5.0). Pharmacodynamic (PD) markers were also assessed, including total IgG and AChR‐Ab levels on Day 1 and at Weeks 4, 14, and 21. In the fixed cycles arm, the Week 14 and Week 21 PD samples were collected before initiation of the treatment cycle.

### 2.4 Statistical Analysis

An analysis of covariance model (ANCOVA) was used to estimate the mean change from baseline in total MG‐ADL score averaged across 21 weeks for each treatment arm, and the two‐sided 95% confidence interval for the difference between the two treatment arms (mean change from baseline in total MG‐ADL score averaged across 21 weeks in the fixed cycles arm and the mean change from baseline in total MG‐ADL score averaged across 21 weeks in the Q2W regimen arm). The model includes treatment arm as a factor and baseline MG‐ADL total score as a covariate to account for any differences in baseline MG‐ADL scores. Additionally, a post hoc mixed model for repeated measures (MMRM) analysis was conducted to assess changes from baseline in total MG‐ADL score over time, with treatment, visit, and treatment by visit interaction as fixed effects, and baseline total MG‐ADL score as a covariate to account for any differences in baseline MG‐ADL scores. Other endpoints were summarized by treatment arm until Week 21.

## 3 Results

### 3.1 Participant Population

**FIGURE 2:**
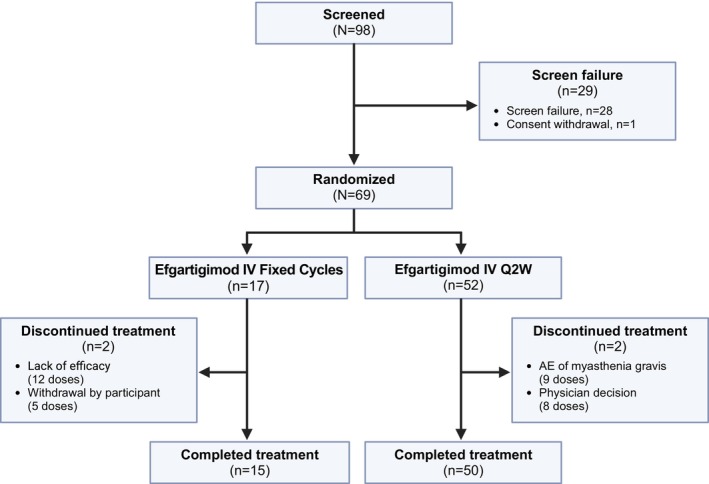
Participant disposition. AE, adverse event; IV, intravenous; Q2W, every other week.

Part A of ADAPT NXT includes data collected up to Week 21 of the study. Of 98 participants screened, 69 were randomly assigned and enrolled (fixed cycles arm, *n* = 17; Q2W arm, *n* = 52; Figure 2). Of the 29 participants who were not enrolled in the study, one withdrew their consent to participate, and 28 did not meet the inclusion criteria of the study.

Participant baseline demographics and clinical characteristics are reported in Table 1, and prior gMG therapies are reported in Table S2. Mean (SD) baseline MG‐ADL total scores were higher in the Q2W arm (9.8 [3.3]) vs. the fixed cycles arm (8.1 [2.2]). Additionally, there were no participants in the fixed cycles arm with a baseline MG‐ADL total score of > 12, whereas 25.0% (*n* = 13/52) of participants in the Q2W had a baseline MG‐ADL total score of > 12. At baseline, 47.1% (*n* = 8/17) of participants in the fixed cycles arm were receiving a NSIST compared with 36.5% (*n* = 19/52) in the Q2W arm.

**TABLE 1:** Baseline demographics and clinical characteristics.

	Efgartigimod IV fixed cycles (*n* = 17)	Efgartigimod IV Q2W (*n* = 52)
Age, years, mean (SD)	52.4 (16.1)	57.1 (16.5)
Age ≥ 65 years, *n* (%)	5 (29.4)	20 (38.5)
Sex, female, *n* (%)	9 (52.9)	34 (65.4)
Race, *n* (%)		
Asian	0	1 (1.9)
Black or African American	2 (11.8)	0
White	14 (82.4)	50 (96.2)
Other	1 (5.9)	0
Unknown	0	1 (1.9)
Time since diagnosis, years, mean (SD)	7.4 (6.6)	6.9 (7.3)
MGFA classification at screening, *n* (%)		
Class II	6 (35.3)	17 (32.7)
Class III	11 (64.7)	33 (63.5)
Class IV	0	2 (3.8)
Total MG‐ADL score, mean (SD)	8.1 (2.2)	9.8 (3.3)
Total MG‐ADL categorization, *n* (%)		
5–12	17 (100.0)	39 (75.0)
> 12	0	13 (25.0)
Previous thymectomy	5 (29.4)	11 (21.2)
Baseline MG therapy, *n* (%)		
Any steroid	10 (58.8)	30 (57.7)
Any NSIST	8 (47.1)	19 (36.5)
Any AChEI	12 (70.6)	49 (94.2)
AChEI only	0	17 (32.7)

Abbreviations: AChEI, acetylcholinesterase inhibitor; IV, intravenous; MG, myasthenia gravis; MG‐ADL, Myasthenia Gravis Activities of Daily Living; MGFA, Myasthenia Gravis Foundation of America; NSIST, nonsteroidal immunosuppressive therapy; Q2W, every other week.

### 3.2 Efficacy

Clinical improvements in MG‐ADL total score were observed across both arms, and the primary endpoint analysis revealed no statistically significant nor clinically meaningful mean differences in the MG‐ADL total score average change from baseline over Weeks 1–21 between the two regimen arms (Table 2). The least squares (LS) mean MG‐ADL total score change in the ANCOVA (using baseline MG‐ADL total score as a covariate) for the fixed cycles arm was −5.1 with a 95% CI of −6.5 to −3.8, whereas it was −4.6 with a 95% CI of −5.4 to −3.8 for the Q2W arm. The LS mean difference between the fixed cycles versus the Q2W arm was −0.5 with a 95% CI of −2.1 to 1.1.

**TABLE 2:** ANCOVA analysis of primary endpoint (mean change from baseline in total MG‐ADL score averaged across 21 weeks).

	Efgartigimod IV fixed cycles		Efgartigimod IV Q2W		Efgartigimod IV fixed cycles vs. Q2W
	*n*	LS mean (95% CI)	*n*	LS mean (95% CI)	LS mean difference (95% CI)
mITT analysis set^a^	17	−5.1 (−6.5 to −3.8)	52	−4.6 (−5.4 to −3.8)	−0.5 (−2.1 to 1.1)

Abbreviations: ANCOVA, analysis of covariance; IV, intravenous; LS, least squares; MG‐ADL, Myasthenia Gravis Activities of Daily Living; mITT, modified intent‐to‐treat; Q2W, every other week.

^a^ The ANCOVA model includes the treatment arm as a factor and the baseline MG‐ADL total score as a covariate. The mITT analysis set was defined as all randomized participants with an MG‐ADL total score at baseline and ≥ 1 postbaseline analysis visit at or before Week 21.

**FIGURE 3:**
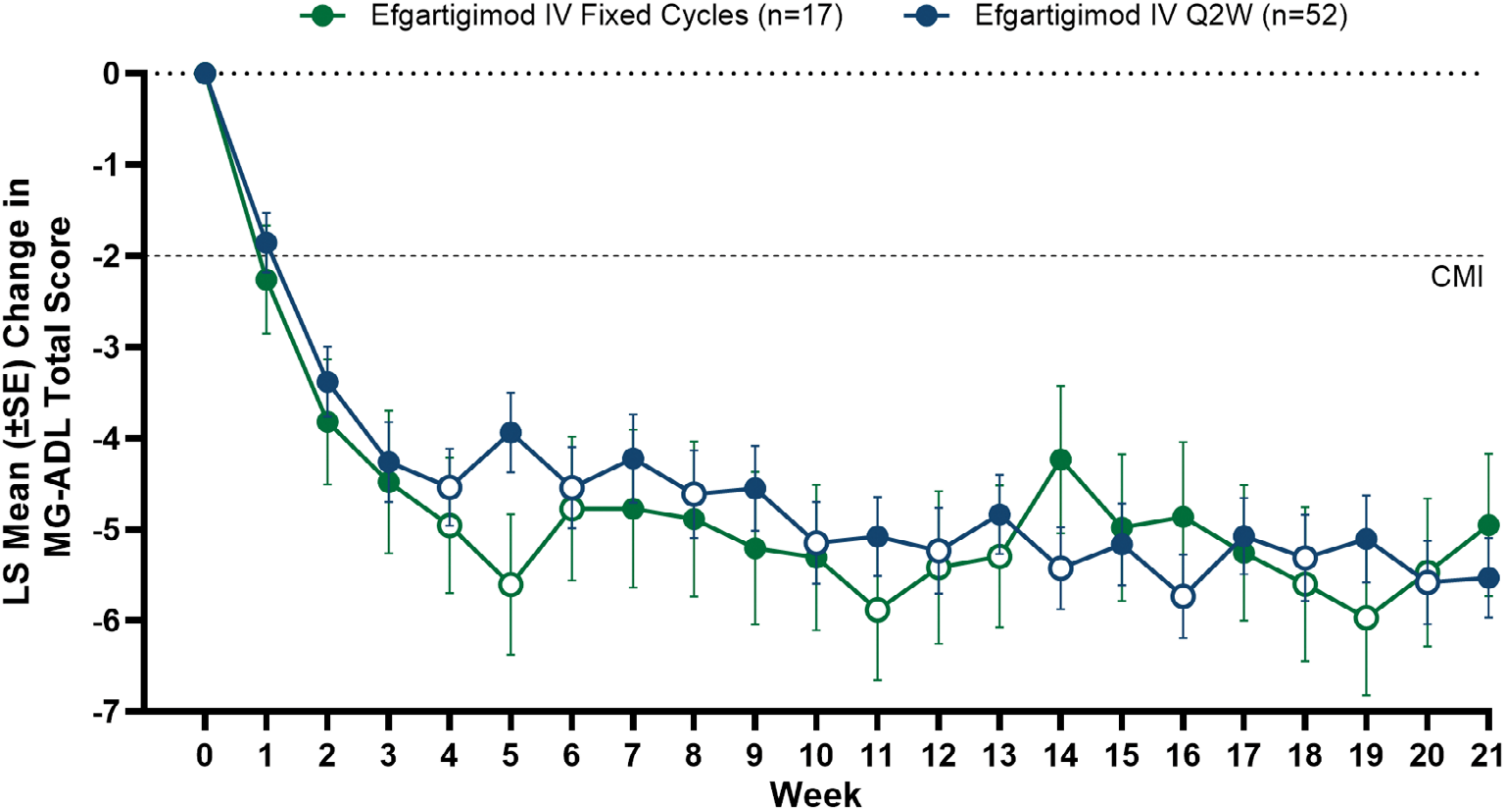
LS mean change from baseline in total MG‐ADL score across Weeks 1–21. Mixed model for repeated measurements analysis with treatment, visit, and treatment by visit interaction as fixed effects, and baseline total MG‐ADL score as a covariate. Solid data points indicate weeks in which efgartigimod was administered and open data points indicate weeks in which efgartigimod was not administered for each respective dosing regimen. CMI, clinically meaningful improvement; IV, intravenous; LS, least squares; MG‐ADL, Myasthenia Gravis Activities of Daily Living; Q2W, every other week.

**FIGURE 4:**
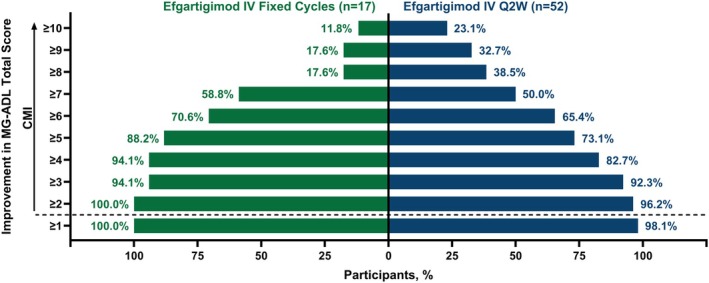
Proportion of participants with increasing MG‐ADL threshold improvement during the study. CMI, clinically meaningful improvement; IV, intravenous; MG‐ADL, Myasthenia Gravis Activities of Daily Living; Q2W, every other week.

Clinical improvements in LS mean MG‐ADL total scores were observed as early as Week 1 across both fixed cycles and Q2W arms. Both arms sustained similar clinically meaningful improvements through Week 21 of the study, and there were no clinically meaningful differences between the 2 dosing regimens in the LS mean MG‐ADL total score changes from baseline at any time point (Figure 3). Additionally, achievement of MSE (an MG‐ADL score of 0–1 at any time during the 21‐week study) was similar between study arms, with 47.1% (*n* = 8/17) and 44.2% (*n* = 23/52) of participants reaching MSE in the fixed cycles and Q2W arms, respectively. Furthermore, participants exhibited substantial improvement in MG‐ADL scores, with 88.2% (*n* = 15/17) of participants in the fixed cycles arm and 73.1% (*n* = 38/52) in the Q2W arm achieving ≥ 5‐point improvement in MG‐ADL total score during the study (Figure 4).

### 3.3 Pharmacodynamics

One week after the fourth administration (Week 4), the mean (SE) percent change from baseline in total IgG was −64.8% (1.9) and −67.6% (1.1) in the fixed cycles and Q2W arms, respectively. In the fixed cycles arm, the mean (SE) percent change from baseline in total IgG levels immediately before initiating the second and third treatment cycles at Weeks 14 and 21 were −38.4% (3.4) and −29.9% (5.8); sampling in this arm for these time points occurred before the initiation of a new cycle, when the smallest reduction of IgG after a treatment cycle would be anticipated. In the Q2W arm, the mean (SE) percent change from baseline in total IgG at Weeks 14 and 21 were −60.4% (4.3) and −58.0% (2.0), respectively. The mean (SE) percent changes from baseline in AChR‐Ab levels over time for both treatment arms are reported in Table S3.

### 3.4 Safety

Efgartigimod IV was well tolerated in both the fixed cycles and Q2W arms (Table 3). The most common (reported by ≥ 10% of total participants) treatment‐emergent adverse events (TEAEs) were COVID‐19 (18.8%, *n* = 13/69), headache (18.8%, *n* = 13/69), and upper respiratory tract infection (10.1%, *n* = 7/69). Most TEAEs were mild to moderate in severity; severe TEAEs occurred in 17.6% (*n* = 3/17) and 13.5% (*n* = 7/52) of participants in the fixed cycles and Q2W arms, respectively. Serious TEAEs occurred in 5.9% (*n* = 1/17) of participants in the fixed cycles arm and in 13.5% (*n* = 7/52) of participants in the Q2W arm. Of the 8 serious TEAEs, 3 were infections (COVID‐19 in the fixed cycles arm; bronchitis and respiratory syncytial virus in the Q2W arm) and none led to study discontinuation or were considered related to treatment. No participants in the fixed cycles arm discontinued the study due to TEAEs, while one participant in the Q2W arm discontinued due to worsening MG. No deaths occurred during the study and no new safety signals emerged compared with previous efgartigimod studies.

**TABLE 3:** Overview of adverse events.

	Efgartigimod IV fixed cycles (*n* = 17, PYFU = 6.9)			Efgartigimod IV Q2W (*n* = 52, PYFU = 20.9)			Efgartigimod IV total population (*n* = 69, PYFU = 27.8)		
	*n*	%	ER^a^	*n*	%	ER^a^	*n*	%	ER^a^
TEAE	16	94.1	12.0	43	82.7	10.1	59	85.5	10.6
Serious TEAE	1	5.9	0.4	7	13.5	0.3	8	11.6	0.4
Grade ≥ 3 TEAE	3	17.6	1.3	7	13.5	0.4	10	14.5	0.6
Fatal TEAE	0	—	—	0	—	—	0	—	—
Discontinued study due to TEAEs^b^	0	—	—	1	1.9	< 0.1	1	1.4	< 0.1
Most frequent TEAEs^c^									
COVID‐19	2	11.8	0.3	11	21.2	0.5	13	18.8	0.5
Headache	5	29.4	1.2	8	15.4	0.6	13	18.8	0.8
Upper respiratory tract infection	2	11.8	0.4	5	9.6	0.4	7	10.1	0.4

Abbreviations: ER, event rate; IV, intravenous; PYFU, participant years of follow‐up; Q2W, every other week; TEAE, treatment‐emergent adverse event.

^a^ ER was calculated as the number of events/PYFU.

^b^ The TEAE that led to study discontinuation in the Q2W arm was myasthenia gravis exacerbation and was not considered related to treatment.

^c^ Reported by ≥ 10% of total participants.

Neither efgartigimod treatment regimen resulted in reductions to serum albumin levels. There were no clinically relevant changes in other laboratory values (including albumin or cholesterol levels), vital signs, or electrocardiogram results.

## 4 Discussion

ADAPT NXT assessed the efficacy, safety, and tolerability of IV efgartigimod administered in two dosing regimens not explicitly included in previous trials of participants with gMG, with the aim to extend options for individualized efgartigimod dosing. Part A of ADAPT NXT demonstrated that both fixed cycles and an initial cycle followed by Q2W dosing resulted in rapid, robust, and clinically meaningful improvements in MG‐ADL scores that were sustained through 21 weeks. Additionally, both regimens of efgartigimod were well tolerated and had a safety profile consistent with previous studies.

In the ADAPT clinical trial, efgartigimod was administered in treatment cycles of 4 once‐weekly infusions with the frequency of cycles based on clinical evaluation; most participants exhibited rapid and clinically meaningful improvements in gMG symptoms across multiple disease‐specific scales [16]. Additionally, 44.6% of AChR‐Ab+ participants treated with efgartigimod achieved MSE at any time during the study (up to 3 cycles) [35]. In the ADAPT+ open‐label extension, long‐term treatment with efgartigimod resulted in consistent and repeatable clinical improvements over multiple cycles [34, 35]. The efficacy of efgartigimod observed during ADAPT NXT is consistent with these previous studies, where clinical improvements in MG‐ADL total score were observed as early as 1 week after efgartigimod administration and were maintained throughout the 21‐week study across both treatment regimens. Importantly, 47.1% and 44.2% of participants in the fixed cycles and Q2W dosing arms, respectively, achieved MSE [16, 35].

The observations from ADAPT NXT have important implications for the utilization of efgartigimod in clinical practice. For instance, these data support one potential approach where the first 3 cycles of efgartigimod could be given at fixed intervals with 4 weeks between cycles, with clinical evaluation of the patient's response used to plan for the individualization of efgartigimod dosing in subsequent cycles (if desirable to the patient and treating physician). This dosing method has been described and employed with success in a clinical setting [36]. Alternatively, for patients who might prefer more regular efgartigimod dosing, the data from ADAPT NXT suggest that an initial cycle of 4 once‐weekly infusions followed by Q2W administration is also effective and well tolerated. These data from ADAPT NXT add to the emerging real‐world experience with efgartigimod in routine clinical practice, where success with both flexible and fixed dosing regimens has been reported [36, 37, 38, 39, 40, 41]. Taken together, these data suggest that after initiating treatment with a fixed cycle, efgartigimod dosing can be highly individualized to the treatment goals of the treating physician and the desires of the patient.

Efgartigimod is well tolerated with individualized cycles, and now additionally across both fixed cycles and Q2W dosing regimens, with no new safety signals compared with previous studies [16, 27, 34]. Most TEAEs were mild to moderate in severity, and only 1 (MG worsening) led to study discontinuation. Three serious infections (COVID‐19, bronchitis, and respiratory syncytial virus) were observed, but none led to study discontinuation or were considered treatment related. Although ADAPT NXT is the first study to evaluate fixed cycles and Q2W efgartigimod dosing regimens in participants with gMG, clinical trials in participants with CIDP and primary ITP have investigated weekly or Q2W administration [24, 25]. In these studies, both weekly and Q2W administration of efgartigimod was well tolerated; adverse events were mostly mild to moderate in severity and did not increase in frequency with additional efgartigimod dosing [24, 25].

Limitations of this study include small numbers of enrolled participants in the fixed cycles arm (due to the 3:1 randomization), lack of a placebo arm, and potential biases in reporting and/or assessment of outcomes due to the open‐label design. While the 21‐week duration of ADAPT NXT Part A is sufficient to assess short‐term outcomes, the ongoing Part B of the study will examine long‐term efficacy and safety, and durability of response. Moreover, this study was not powered to assess which participants may have the most benefit from a particular dosing regimen. Additionally, while there were baseline differences between the two arms in total MG‐ADL score, ANCOVA and MMRM models (in which baseline total MG‐ADL score was controlled for as a covariate) demonstrated similar improvements across both study arms. Likewise, despite modest differences in baseline NSIST use between study arms, similar MG‐ADL improvements were observed. Finally, the SC formulation of efgartigimod was not investigated in ADAPT NXT because, at the time the study was designed, it had not yet received regulatory approval and noninferiority of SC and IV efgartigimod had not yet been established.

In conclusion, patients with gMG receiving efgartigimod in either fixed cycles or Q2W demonstrated rapid and robust improvements in symptoms and function that were sustained over the duration of the 21‐week treatment period. From the perspective of both the patient and treating physician, having multiple options and flexibility regarding efgartigimod dosing provides the opportunity to further tailor treatment plans to individual patients [8, 42, 43, 44]. The efficacy and safety demonstrated in ADAPT NXT build upon previous studies and provide additional evidence supporting individualized efgartigimod dosing options to maximize and maintain clinical efficacy in patients with gMG.

## Author Contributions

Concept and design: A.A.H., K.G.C., V.B., Y.H., K.G., G.S., E.C.‐V., E.B., D.G., A.S., D.H., R.M., A.M., S.A. Data collection: A.A.H., K.G.C., V.B., Y.H., K.G., G.S., E.C.‐V., R.M., A.M., S.A. Statistical analysis: E.B., A.S., R.H.J., D.H., D.M. Interpretation of results: A.A.H., K.G.C., V.B., Y.H., K.G., G.S., E.C.‐V., E.B., D.G., A.S., R.H.J., D.H., D.M., R.M., A.M., S.A. Manuscript preparation: A.A.H., E.B., D.G., A.S., R.H.J., D.H., D.M. Critical review of manuscript: A.A.H., K.G.C., V.B., Y.H., K.G., G.S., E.C.‐V., E.B., D.G., A.S., R.H.J., D.H., D.M., R.M., A.M., S.A. Study supervision: R.M., A.M.

## Conflicts of Interest

A.A.H. has received research support from argenx, Alexion Pharmaceuticals Inc., VielaBio, UCB Pharma, Genentech, Regeneron, and Sanofi. He has received consulting fees from argenx, Alexion Pharmaceuticals Inc., and UCB. K.G.C. has received consulting fees for advisory boards and/or received speaker honoraria from Alexion Pharmaceuticals Inc., Alnylam, Amicus, argenx, Biogen, CSL Behring, Ipsen, Janssen, Lupin, Pfizer, Roche, Sanofi‐Genzyme, and UCB. K.G.C. is the chairholder of the Emil von Behring Chair for Neuromuscular and Neurodegenerative Disorders by CSL Behring. V.B. has received research support from AZ‐Alexion, Grifols, CSL, UCB, argenx, Takeda, Octapharma, Akcea, Momenta (J&J), Immunovant, Ionis, and Viela. Y.H. has no disclosures to report. K.G. has received consulting/speaking honoraria from Alexion Pharmaceuticals Inc., and argenx, and consulting honoraria from UCB and Amgen. G.S. has received consulting fees/honoraria or support for meeting participation from Alexion Pharmaceuticals Inc., argenx, UCB, Immunovant Inc., and Biogen Inc. E.C.‐V. has received consulting/speaker fees from argenx, UCB, Alexion Pharmaceuticals Inc., and Janssen. E.B., D.G., A.S., R.H.J., D.H., and D.M. are employees of argenx. R.M. has received consulting fees/honoraria or support for meeting participation from Alexion Pharmaceuticals Inc., argenx, Ra Pharmaceuticals, Biomarin, Catalyst, UCB, TEVA, Merck, Roche, Janssen, and Biogen Inc. A.M. received speaker honoraria from Alexion Pharmaceuticals Inc., argenx, Grifols, SA, and Hormosan Pharma GmbH; honoraria from argenx, Alexion Pharmaceuticals Inc., UCB, Janssen, and Merck for consulting services; and financial research support (paid to his institution) from Octapharma, argenx, and Alexion Pharmaceuticals Inc. He is a member of the medical advisory board of the German Myasthenia Gravis Society. S.A. received speaker honoraria from Alexion Pharmaceuticals Inc., argenx, Sanofi, Pfizer, and LFB and honoraria from Alexion Pharmaceuticals Inc., UCB, Janssen, Sanofi, Pfizer, Biogen Inc., and LFB for consulting services.

## Supporting information


Data S1.

## Data Availability

argenx is committed to responsible data sharing regarding the clinical trials they fund. Included in this commitment is access to anonymized, individual, and trial‐level data (analysis datasets), and other information (e.g., protocols and clinical study reports), as long as the trials are not part of an ongoing or planned regulatory submission. This includes requests for clinical trial data for unlicensed products and indications. These clinical trial data can be requested by qualified researchers who engage in rigorous independent scientific research and will only be provided after review and approval of a research proposal and statistical analysis plan and execution of a data sharing agreement. Data requests can be submitted at any time, and the data will be accessible for 12 months. Requests can be submitted to esr@argenx.com.
