# Supplementary material for: ADAPT NXT: Fixed Cycles or Every‐Other‐Week IV Efgartigimod in Generalized Myasthenia Gravis
Source: Ann Clin Transl Neurol. 2025 Apr 14;12(6):1162–70. doi: 10.1002/acn3.70051 (PMC12172114; doi:10.1002/acn3.70051)
Supplement: Supplementary file 1 — Data S1. [file ACN3-12-1162-s001.docx]

**Supporting Information**

For the original article: **ADAPT NXT: Fixed Cycles or Every-Other-Week IV Efgartigimod in Generalized Myasthenia Gravis**

**Supplemental Table 1. Full inclusion and exclusion criteria for study entry**

| **Inclusion Criteria** | Participants are eligible to be included in the study only if all of the following criteria apply:   1. Capable of giving signed informed consent, which includes compliance with the requirements and restrictions listed in the informed consent form. 2. At least 18 years of age at the time of signing the informed consent. 3. Diagnosed with gMG with confirmed documentation and supported by a physical exam and confirmed seropositivity for AChR-Abs. 4. Meets the clinical criteria as defined by the MFGA class II, III, or IV. 5. Has an MG-ADL total score ≥5 at screening and the Day 1 visit, with more than 50% of the score due to nonocular symptoms. 6. Concomitant gMG treatment is permitted. Permitted concomitant gMG treatment includes NSISTs, steroids, and/or AChE inhibitors. If receiving corticosteroids and/or NSISTs, must be on a stable dose for at least 1 month before screening. 7. Contraceptive use by men and women should be consistent with local regulations regarding the methods of contraception for those participating in clinical studies.    1. **Male participants:** are not allowed to donate sperm from the time of signing the informed consent form until the end of the study.    2. **Female participants:** WOCBP must have a negative serum pregnancy test at screening, and negative urine pregnancy test at baseline before efgartigimod can be administered. WOCBP must use a highly effective or acceptable contraception method, which should be maintained at minimum until 90 days after last dose of efgartigimod. |
| --- | --- |
| **Exclusion Criteria** | Participants are excluded from the study if any of the following criteria apply:   1. Clinically significant uncontrolled active or chronic bacterial, viral, or fungal infection at screening. 2. A positive test for SARS-CoV-2 at screening. 3. Any other known autoimmune disease that, in the opinion of the investigator, would interfere with an accurate assessment of the clinical symptoms of gMG and/or put the participant at undue risk. 4. History of malignancy unless deemed cured by adequate treatment with no evidence of reoccurrence for ≥3 years before the first administration of efgartigimod. Participants with the following cancers can be included at any time, provided they are adequately treated at screening: basal cell or squamous cell skin cancer, carcinoma in situ of the cervix or breast, incidental histological finding of prostate cancer (TNM stage T1a or T1b). 5. Clinical evidence of other significant serious diseases, a recent (<3 months) major surgery, or any other condition that, in the opinion of the investigator, could confound the results of the study or put the participant at undue risk. 6. A thymectomy within 3 months of screening. 7. Pregnant or lactating females and those who intend to become pregnant during the study or within 90 days of the last dose of efgartigimod. 8. Use of the following prior or concomitant therapies:    1. IVIg or subcutaneous (SC)Ig within 14 days of Day 1.    2. Rituximab within 6 months of Day 1.    3. Eculizumab within 1 month of Day 1.    4. Other monoclonal antibodies (eg, adalimumab, tocilizumab, ixekizumab) within 5 half-lives of the monoclonal antibodies before Day 1.    5. Use of any other investigational product within 3 months or 5 half-lives, whichever is longer, before Day 1.    6. Receipt of a live or live-attenuated vaccine within 4 weeks of screening. The receipt of any inactivated, subunit, polysaccharide, conjugate vaccine at any time before screening is not considered exclusionary. 9. Previous participation in a clinical study or patient access program during which they were treated with efgartigimod. 10. Positive serum test at screening for an active viral infection with any of the following conditions: HBV that is indictive of an acute or chronic infection, HCV based on HCV antibody assay (unless associated with a negative HCV RNA test), HIV based on test results that are associated with an AIDS-defining condition or a CD4 count ≤200 cells/mm.^3^ 11. Total IgG <6 g/L at screening. 12. Known hypersensitivity reaction to efgartigimod or any of its excipients. 13. The participant stands in any relationship of dependency with the sponsor. 14. The participant has been institutionalized due to an official or judicial order. |

AChE, acetylcholinesterase; AChR-Ab; acetylcholine receptor antibody; AIDS, acquired immunodeficiency syndrome; CD4, cluster of differentiation 4; gMG; generalized myasthenia gravis; HBV, hepatitis B virus; HCV, hepatitis C virus; HIV, human immunodeficiency virus; Ig, immunoglobulin; IV, intravenous; MG-ADL, Myasthenia Gravis Activities of Daily Living; MGFA, Myasthenia Gravis Foundation of America; NSIST, nonsteroidal immunosuppressive therapies; RNA, ribonucleic acid; SARS-CoV-2, severe acute respiratory syndrome coronavirus 2; SC, subcutaneous; TNM, tumor, node, and metastasis; WOCBP, women of child bearing potential.

**Supplemental Table 2. gMG therapies started before the first efgartigimod infusion and could have been stopped or were ongoing at time of the study**

|  | **Efgartigimod IV**  **Fixed Cycles**  **(n=17)** | **Efgartigimod IV**  **Q2W**  **(n=52)** |
| --- | --- | --- |
| **gMG therapy ,** n (%) |  |  |
| ≥1 prior gMG therapy | 15 (88.2) | 52 (100.0) |
| ≥2 prior gMG therapy | 14 (82.4) | 37 (71.2) |
| ≥3 prior gMG therapy | 6 (35.3) | 25 (48.1) |
| **NSISTs,** n (%) |  |  |
| ≥1 prior NSIST | 8 (47.1) | 23 (44.2) |
| ≥2 prior NSIST | 0 | 2 (3.8) |
| ≥3 prior NSIST | 0 | 0 |
| **MG therapy type,**n (%) |  |  |
| Acetylcholine chloride | 0 | 1 (1.9) |
| Ambenonium chloride | 1 (5.9) | 2 (3.8) |
| Azathioprine | 3 (17.6) | 11 (21.2) |
| Azathioprine sodium | 1 (5.9) | 1 (1.9) |
| Ciclosporin | 0 | 1 (1.9) |
| Cortisone acetate | 1 (5.9) | 0 |
| Deflazacort | 1 (5.9) | 0 |
| Eculizumab | 1 (5.9) | 2 (3.8) |
| Hydrocortisone | 0 | 1 (1.9) |
| Immunoglobulin G human | 1 (5.9) | 0 |
| Immunoglobulin human normal | 2 (11.8) | 5 (9.6) |
| Immunoglobulins | 0 | 1 (1.9) |
| Immunoglobulins (not otherwise specified) | 1 (5.9) | 12 (23.1) |
| Methotrexate | 1 (5.9) | 2 (3.8) |
| Methylprednisolone | 1 (5.9) | 4 (7.7) |
| Mycophenolate mofetil | 3 (17.6) | 7 (13.5) |
| Neostigmine bromide | 0 | 1 (1.9) |
| Prednisolone | 2 (11.8) | 3 (5.8) |
| Prednisone | 8 (47.1) | 25 (48.1) |
| Pyridostigmine^a^ | 3 (17.6) | 12 (23.1) |
| Pyridostigmine bromide | 9 (52.9) | 41 (78.8) |
| Tacrolimus | 0 | 3 (5.8) |

^a^Treatment with “pyridostigmine” was erroneously captured in the database; the only authorized marketed form is pyridostigmine bromide. gMG, generalized myasthenia gravis; IV, intravenous; MG, myasthenia gravis; NSIST, nonsteroidal immunosuppressive therapy.

**Supplemental Table 3. Percentage changes from baseline in total IgG and AChR-Ab levels over time by treatment arm**^a^

|  | **Efgartigimod IV Fixed Cycles**  **(n=17)** | | | | **Efgartigimod IV Q2W**  **(n=52)** | | | | |
| --- | --- | --- | --- | --- | --- | --- | --- | --- | --- |
|  | **Total IgG** | | **AChR-Ab** | | **Total IgG** | | **AChR-Ab** | | |
| **Visit** | n | Mean (SE) | n | Mean (SE) | n | Mean (SE) | n | Mean (SE) |  |
| Week 4 | 17 | -64.8 (1.9) | 17 | -52.7 (4.1) | 46 | -67.6 (1.1) | 44 | -58.8 (1.8) |  |
| Week 14 | 14 | -38.4 (3.4)^b^ | 15 | -21.6 (3.8)^b^ | 49 | -60.4 (4.3)^c, d^ | 46 | -54.2 (2.9)^c^ |  |
| Week 21 | 14 | -29.9 (5.8)^b^ | 14 | -13.2 (4.4)^b^ | 48 | -58.0 (2.0)^d, e^ | 46 | -45.0 (3.0)^e^ |  |

^a^Sample size for total IgG and AChR-ab levels may differ due to protocol deviations or technical issues limiting sample collection or analysis. ^b^PD samples for Weeks 14 and 21 in the fixed cycles arm were collected before initiation of the treatment cycle, when the smallest reduction of IgG after a treatment cycle would be anticipated. The long-term extension studies ADAPT+ and ADAPT-SC+ demonstrated that reductions in total IgG, IgG subtypes, and AChR antibodies were repeatable across multiple treatment cycles^1,2^. ^c^In the Q2W arm, the Week 14 PD sample was collected one week after the last efgartigimod infusion. ^d^Weekly or Q2W efgartigimod dosing has been studied in CIDP and ITP and these clinical trials showed that total IgG levels remained consistently reduced across the course of both studies^3,4^. ^e^In the Q2W arm, the Week 21 PD sample was collected two weeks after the last efgartigimod infusion. AChR, acetylcholine receptor; AChR-Ab; acetylcholine receptor antibody; CIDP, chronic inflammatory demyelinating polyneuropathy; IgG, immunoglobulin G; ITP, primary immune thrombocytopenia; IV, intravenous; PD, pharmacodynamic; Q2W, every other week.

**Supplemental Table 4. ADAPT NXT Study Group investigators and sites**

| **Investigator** | **Site** | **Country** |
| --- | --- | --- |
| Ali Habib  Tahseen Mozzaffar Jeffrey Mullen Lauren Harris Isela Hernandez | University of California, Irvine | United States |
| Arjun Seth  Fang Sun  Robert Kalb  Erik Pioro | Northwestern University Feinberg School of Medicine | United States |
| Mamatha Pasnoor  Mazen Dimachkie  Jeffrey Statland  Duaa Jabari  Omar Jawdat  Constatine Farmakidis  Sandhya Sasidharan  Melissa Hayes | University of Kansas Medical Center | United States |
| Marc Feinberg  Scott Blumenthal  Frederick Boltz  Joannes Paul  Gabrielle Demaria  Monique Tromp | South Florida Neurology Associates P.A. | United States |
| Gregory Sahagian  Benjamin Frishberg  Tara Quesnell  Kinjal Madhav  John Heinen  Andrew Bierman  April Tenorio  Elizabeth Rice  Jisoo Lee  Nicole Megali | The Neurology Center of Southern California | United States |
| Andrew Gordon  Akash Patel  Lisa Jackson  Joanna Kaleta  Ahmir Khan | Northwest Neurology, Ltd. | United States |
| Michael Rivner  Benjamin Barnes | Augusta University | United States |
| Kelly Gwathmey  Albert Smith  Neel Dixit  Kathryn Gallanosa  Amanda Butler | Virginia Commonwealth University | United States |
| Yessar Hussain  Hannah Machemehl  Casey Kafena  Shailesh Reddy  Mary Trunk  Stephanie Gonsoulin | Austin Neuromuscular Center | United States |
| Sami Khella  Thomas Keenan  Swathii Vijayaraghavan | University of Pennsylvania, School of Medicine | United States |
| Noona Leavell  Anson Wilks | Oregon Health & Science University | United States |
| Sonia Kalirao  Donna Luke  Anthony Shydohub | Healthcare Innovations Institute LLC | United States |
| Oliver Blanchard  Angela Genge  Jeanne Teitelbaum  Raquel Farias  Smita Patel  Priya Nagpal  Rami Massie | McGill University, Montreal Neurological Hospital | Canada |
| Michael Nicolle  Christen Shoesmith  Denise Hulley | London Health Sciences Center | Canada |
| Anneke van der Kooi Filip Eftimov | Amsterdam UMC | The Netherlands |
| Kristl Claeys  Bram De Wel  Philip Van Damme  Matthias Opsomer | University Hospitals Leuven | Belgium |
| Shahram Attarian  Aude Marie Grapperon  Ludivine Kouton  Emmanuelle Salort-Campana  Emilien Delmont | Reference Center for Neuromuscular Diseases and ALS, Clinical Neuroscience Center - La Timone University Hospital | France |
| Sophie Demeret  Giorgia Querin  Clemence Marois  Camille Giron  Anthony Behin | Groupe Hospitalier Pitié-Salpétriére | France |
| Sabrina Sacconi  Angela Puma  Michele Cavalli  Luisa Villa  Andra Ezaru | CHU de Nice | France |
| Celine Tard  Jean-Baptiste Davion | CHRU de Lille - Hôpital Roger Salengro | France |
| Guilhem Sole  Fanny Duval  Diane Beauvais | CHU de Bordeaux - Hôpital Pellegrin | France |
| Elena Cortes-Vincente  David Reyes  Ana Vesperinas | Hospital de la Santa Creu i Sant Pau | Spain |
| Julio Pardo Fernandez  Tania Garcia Sobrino  Esperanza Ortegon | Complejo Hospitalario Universitario de Santiago de Compostela (C.H.U.S.) | Spain |
| Raffaele Iorio  Lucia Campetella  Eleonora Sabatelli  Sofia Marini  Silvia Falso  Martina Marini | Fondazione Policlinico Universitario “A. Gemelli” IRCCS | Italy |
| Renato Mantegazza  Carlo Antozzi  Fiametta Vanoli  Lorenzo Maggi  Silvia Bonnano  Annamaria Gallone  Rita Frangiamore | Fondazione IRCCS Istituto Neurologico Carlo Besta | Italy |
| Marina Grandis  Elena Scarsi  Sara Massucco  Margherita Bellucci  Elena Faedo | Università degli Studi di Genova | Italy |
| Rocco Liguori  Maria Pia Giannoccaro  Veria Vaachiano  Claudia Faini  Alessandro Furia | Alma Mater Studiorum -Università di Bologna | Italy |
| Gabriele Siciliano  Erika Schirinzi  Michelangelo Maestri Tassoni  Ester Latini  Melania Guida | Azienda Ospedaliero  Universitaria Pisana University of Pisa | Italy |
| Fritz Zimprich Fiona Jaeger  Martin Krenn  Jakob Rath  Rosa Weng  Gudrun Zulehner | AKH Wien, Universitätsklinikum | Austria |
| Wolfgang Loescher  Julia Wanschitz  Anna Hotter  Corinne Horlings | Univ.-Klinik für Neurologie, Medizinische Universität Innsbruck | Austria |
| Urszula Chyrchel-Paszkiewicz  Magdalena Ogórek Magdalena Kozłowska | Prywatny Gabinet Lekarski Urszula Chyrchel-Paszkiewicz/Clinirem Sp z o.o. | Poland |
| Krzysztof Banaszkiewicz  Ewa Majcherczyk  Krystian Majkowski  Kacper Szewczyk | Michalski i Partnerzy Lekarze Spka Partnerska ul | Poland |
| Tim Hagenacker  Benjamin Stolte  Svenja Brakemeier | Universitätsklinikum Essen | Germany |
| Christiane Schneider- Gold Kalliopi Pitarokoili  Jeremias Motte | Dept. of Neurology, St. Josef Hospital, Ruhr University Bochum | Germany |
| Andreas Meisel  Sarah Hoffman  Hannah Pressler  Frauke Stascheit  Sophie Lehnerer  Maike Stein  Meret Herdick  Paolo Doksani | Charité - Universitätsmedizin Berlin | Germany |
| Thomas Skripuletz  Kurt-Wolfram Sühs  Philipp Schwenkenbecher  Lea Grote-Levi  Tabea Seeliger  Nora Möhn  Martin Hümmert  Sandra Nay | Medzinische Hochschule Hannover | Germany |
| Temur Margania  Dali Kankava | Ltd. “New Hospitals” | Georgia |
| Roman Shakarishvili  Nana Kvirkvelia  Elene Nebadze | Ltd. Petre Sarajishvili Institute of Neurology | Georgia |
| Alexander Tsiskaridze  Tamar Vashadze | Ltd. "Pineo Medical Ecosystem" | Georgia |

**Supplemental References**

1. Howard JF, Jr, Bril V, Vu T, et al. Long-term safety, tolerability, and efficacy of efgartigimod (ADAPT+): interim results from a phase 3 open-label extension study in participants with generalized myasthenia gravis. Front Neurol. 2024;14:1284444.

2. Howard JF, Jr., Vu T, Li G, et al. Subcutaneous efgartigimod PH20 in generalized myasthenia gravis: A phase 3 randomized noninferiority study (ADAPT-SC) and interim analyses of a long-term open-label extension study (ADAPT-SC+). Neurotherapeutics. 2024;21(5):e00378.

3. Broome CM, McDonald V, Miyakawa Y, et al. Efficacy and safety of the neonatal Fc receptor inhibitor efgartigimod in adults with primary immune thrombocytopenia (ADVANCE IV): a multicentre, randomised, placebo-controlled, phase 3 trial. Lancet. 2023;402(10413):1648-1659.

4. Allen JA, Lin J, Basta I, et al. Safety, tolerability, and efficacy of subcutaneous efgartigimod in patients with chronic inflammatory demyelinating polyradiculoneuropathy (ADHERE): a multicentre, randomised-withdrawal, double-blind, placebo-controlled, phase 2 trial. Lancet Neurol. 2024;23(10):1013-1024.
